# Insect–Antioxidants Symbiotic Nexus—Pathway for Sustainable and Resilient Aquaculture: A Case Study for Evaluating Koi Carp Growth and Oxidative Stress Status

**DOI:** 10.3390/antiox14040371

**Published:** 2025-03-21

**Authors:** Alina Antache, Ira-Adeline Simionov, Ștefan-Mihai Petrea, Aurelia Nica, Puiu-Lucian Georgescu, Lăcrămioara Oprică, Marius-Nicușor Grigore, Mircea Oroian, Daniela Jitaru, Andreea Liteanu, Alin-Stelian Ciobîcă, Vladimir Poroch

**Affiliations:** 1 Department of Food Science, Food Engineering, Biotechnology and Aquaculture, Faculty of Food Science and Engineering, “Dunărea de Jos” University of Galați, 800008 Galați, Romania; alina.antache@ugal.ro (A.A.); stefan.petrea@ugal.ro (Ș.-M.P.); aurelia.nica@ugal.ro (A.N.); 2 Rexdan Research Infrastructure, “Dunărea de Jos” University of Galați, 800008 Galați, Romania; 3 Department of Biology, Faculty of Biology, Alexandru Ioan Cuza University of Iasi, Bd. Carol I no. 20A, 700505 Iasi, Romania; lacramioara.oprica@uaic.ro (L.O.); alin.ciobica@uaic.ro (A.-S.C.); 4 Department of Chemistry, Physics and Environment, Faculty of Science and Environment, “Dunărea de Jos” University of Galați, 800008 Galați, Romania; 5 Doctoral School of Biology, Alexandru Ioan Cuza University of Iasi, Bd. Carol I no. 20A, 700505 Iasi, Romania; nicusor.grigore@uaic.ro; 6 Faculty of Food Engineering, “Ștefan cel Mare” University of Suceava, 720229 Suceava, Romania; m.oroian@fia.usv.ro; 7 Department of Hematology, Regional Institute of Oncology, 700483 Iași, Romania; danielajitaru@yahoo.com (D.J.); andreeaberbece@yahoo.com (A.L.); 8 Centre of Biomedical Research, Romanian Academy, Bd. Carol I, no. 8, 700506 Iasi, Romania; 9 Academy of Romanian Scientists, Str. Splaiul Independentei no. 54, Sector 5, 050094 Bucharest, Romania; 10 “Ioan Haulica” Institute, Apollonia University, Păcurari Street 11, 700511 Iasi, Romania; 11 Department of Medicine III, Faculty of Medicine, “Grigore T Popa” University of Medicine and Pharmacy, 700111 Iași, Romania; vladimir.poroch@umfiasi.ro

**Keywords:** functional fish feed, beetroot, turmeric, oxidative stress, antioxidants

## Abstract

Various innovative fish feeds were tested for the production of koi carp in a recirculating aquaculture system, considering insect meal (*Acheta domestica*) as the main protein source and phytogenic additives (*Curcuma longa*—turmeric and *Beta vulgaris*—beetroot) as antioxidants, in the spirit of sustainable aquaculture practice. The growth performance, metabolic rate (respirometry), hematological profile, blood biochemical indicators, and oxidative stress of koi carp were determined, using feeds according to the following experimental design: CF—commercial feed, IF—innovative feed based on cricket meal, BIF—innovative feed (IF) with beetroot, and TIF—innovative feed (IF) with turmeric. The TIF recorded the best growth rate. The lowest values of lipid peroxidation (MDA), standard metabolic rate (SMR), and routine metabolic rate (RMR) were registered for the IF and TIF variants. A reduction in MDA was noted, correlated to the decrease in the metabolic rate regarding SMR and RMR for the IF and TIF. An intensification in amylase was recorded in the TIF and BIF. Compared with the CF, it seems that the IF, TIF, and BIF had a beneficial effect on the koi carp by reducing cholesterol, HDL cholesterol, alanine aminotransferase, triglycerides, and urea and by increasing the concentration of calcium and growth hormone in the blood plasma.

## 1. Introduction

The market of ornamental fish has intensified in the last decade, and koi carp (*Cyprinus carpio* var. *koi*) is the species most sold [1]. This can be attributed to the ability of koi carp to adapt and tolerate different conditions within aquatic environments and ecosystems [2]. It is expected that the koi carp market will reach a market value of USD 6.61 billion by 2032 [3]. The koi carp market includes breeding, rearing, and the development of associated products such as equipment, feeds, and water treatment systems [1]. The economic importance of koi fish is due to its cultural background and vibrant coloration; thus, maintaining the physiological immunity of its biomass is a priority for aquaculture engineers [4,5].

The increasing demand for koi carp calls for ensuring the sustainable intensification of the rearing process. In the fish rearing process, a significant part of the costs is related to fish feeds due to the high costs of protein-based ingredients such as fishmeal (FM) [6,7,8,9]. It has been estimated that 68% of the total cost of aquaculture production is represented by the feed input [10]. Besides the high costs, the use of FM in aquaculture feeds raises environmental concerns due to the production of FM having a direct impact on biodiversity [11,12].

In recent years, attempts have been made to replace animal protein from fish feeds with vegetable protein from soybean, sorghum, or wheat, as well as oil from sunflower, rapeseed, and palm. However, it has been highlighted that the total replacement of fish meal in feed leads to a low fish growth performance due to the absence of essential amino acids, as well as a reduced omega-3 fatty acid content [13].

Therefore, FM should be replaced with alternative and sustainable protein sources. Emerging protein sources for fish feeds such as insect meal can be used as a total substitute for FM due to their high protein content (60–80% in dry weight) and high lipid content (31–43%) [14,15,16,17,18]. Insects represent a viable alternative source to FM because their rearing generates a lower environmental impact, and, at the same time, they constitute a natural component of the fish diet [19,20,21,22,23].

Insect-fed animal products have a series of nutritional advantages, such as a high content of essential amino acids, omega-3 fatty acids, and minerals [18,20,22,23]. The annual production of insects in Europe accounts for roughly 6000 tons, for the feed sector alone, and it is estimated to grow 24.4% per year over the next decade [24]. The most used insect species for producing insect meal are crickets (*Acheta domesticus*), houseflies (*Musca domestica*), black soldier flies (*Hermetia illucens*), and mealworms (*Tenebrio molitor*) [25,26,27]. In terms of proximate composition, cricket meal has been highlighted to be the most suitable for aqua feeds, due to its balance between protein (above 50%) and lipid content (below 20%) [28], while other insect meals such as black soldier fly or mealworm contain a high amount of lipids (above 30%), which require a costly defattening process [27,28]. Besides its nutritional value, it has been confirmed that the introduction of cricket meal in fish diets has the potential to enhance immunity and increase the antioxidant response in fish biomass [29]. Some authors have argued that the use of cricket meal in fish feeds may be limited due to the chitin content, which can decrease feed digestibility [30]. Even so, it was observed that in small concentrations, chitin from cricket meal can lead to the immunomodulation of aquatic organisms due to its antioxidant, antimicrobial, and immunostimulatory potential [31,32,33,34]. Owens et al. in 2024 showed that the *Acheta domesticus* meal is a promising alternative protein source for salmonid feeds [35]. Other researchers have concluded that the total replacement of FM with the *Acheta domesticus* meal in the diet of *P. reticulata* species did not generate negative effects on their growth or breeding performance [36]. The replacement of FM by 60% *Acheta domesticus* meal in the diet of red hybrid tilapia (*Oreochromis* sp.) led to a higher growth rate and survival rate compared to those on commercial feed [37].

Several studies have investigated the use of cricket meal in aquafeeds, and it has been shown to positively influence the growth and nutrient utilization of African catfish [32,38].

Considering that ornamental fish rearing needs to be realised harmoniously while maintaining the welfare of fish, the supplementation of feeds with various additives with antioxidant capacities has been proposed [39,40,41]. The supplementation of fish feeds with different concentrations of phytogenic additives such as turmeric, ginger, garlic, lemon, black pepper, basil, beetroot, sea buckthorn, and even banana flowers was demonstrated to enhance fish growth (through the intensification of digestive enzymatic activity) and immunity (through antioxidant effects) [42,43,44,45,46,47]. This is due to their antioxidant properties and capacity to improve growth performance. For instance, turmeric has antimicrobial, antioxidant, anti-inflammatory, appetite-increasing, immunomodulatory, and gastro-protective effects on animal health [48]. Also, beetroot contains betalains, phenols, vitamin B (B1, B2, B6, and B12), carotenoids, folate, and minerals that impart antioxidant activity, including the inhibition of lipid peroxidation [49]. The antioxidant capacity of turmeric is due to the curcumin content of this plant. It was highlighted that the use of curcumin in the diet of *Cyprinus carpio* in a concentration of 15 g/kg feed significantly improved its growth performance, skin mucosal immunity, and serum antioxidant parameters (catalase and superoxide dismutase) [50]. At the same time, it was observed that the use of turmeric (meal or extract) in fish diets promotes gonad development and digestive enzymes [51]. Also, in case of *Oreochomis niloticus*, it was found that turmeric improved the fish’s survival rate [52]. Further, Anene et al. in 2022 showed that the turmeric powder used in the diet of *Clarias gariepinus* did not generate an adverse effect on the physiological responses of the fish [53]. Besides turmeric, the positive effect of *Beta vulgaris* used as a fish feed additive, in different concentrations, on fish growth performance indicators and welfare was observed. In a study conducted over 8 weeks, during which a 2% concentration of *B. vulgaris* leaf powder was administered to *C. carpio*, a higher growth performance was registered [54]. Other studies have shown that the addition of beetroot in the diet of common carp generated a positive effect on its growth [55,56], survival index [55], and blood physiological parameters [56].

Even though the use of house crickets in fish diets has been investigated previously by the scientific community, there is a knowledge gap on how to enhance the antioxidant capacity of this type of feed. Exploring the pairing of *Acheta domesticus* meal with different antioxidant phytogenic additives has become a necessity, as well as an opportunity. Considering this and the European Green Deal strategy, which targets the transition of the European Union’s (EU) economy towards a sustainable one [57], especially through restoring biodiversity and developing solutions for environmentally friendly food production systems, the following study objectives are outlined:

(a) Develop innovative fish feeds for the production of koi carp in a recirculating aquaculture system, based on alternative protein sources (insect flour) and phytogenic additives (*Curcuma longa*—turmeric and *Beta vulgaris*—beetroot) as antioxidants;
(b) Evaluate and characterize the innovative feeds in regards to the amino acid content;
(c) Conduct a comparative analysis to evaluate the growth performance, hematological and biochemical profile, and metabolic rate of the experimental fish biomass.

## 2. Materials and Methods

### 2.1. Ethics Statement

The experiment conducted in the present study was carried out according to the European Directive 2010/63/EU, which regulates the protection of animals used for scientific purposes. The experiment was also approved by the Ethics Committee from ”Dunarea de Jos” University of Galati (no. 6) on 21 April 2023. The experimental tests were conducted by minimizing fish distress, and fish were handled only at the beginning and end of the experimental period. Before blood sampling and biometric measurements, fish were anesthetized. Before performing the experimental tests (24 h prior), no feed was administered to the fish biomass. No specimens were sacrificed within the present study.

### 2.2. Experimental Design

The biological material used in this study was represented by the ornamental koi carp specimen (*Cyprinus carpio* var. koi) with an initial body weight of 73.6 ± 0.59 g/fish. The fish specimens were obtained through artificial reproduction at the Department of Food Science, Food Engineering, Biotechnology, and Aquaculture from “Dunărea de Jos” University of Galati—Romania. The fish were distributed in 12 growth units (in triplicate), and the experiment was conducted for 54 days. The experimental period of 8 weeks was chosen based on consulting the literature [58,59,60,61]. Also, in a study in which fish meal was completely replaced with cricket meal in Nile tilapia diet, the duration of the experiment was 42 days [62]. Fish were previously acclimatized to the recirculating aquaculture pilot systems (RASs) within the Food Science, Food Engineering, Biotechnology, and Aquaculture Department—“Dunărea de Jos” University of Galați, applying a stocking density in each rearing unit of 6.69 ± 0.05 kg/m^3^. The pilot RAS system is composed of rectangular fish rearing units (80 × 48 × 48 cm), connected to a mechanical filtration unit (multiple sizes of filtration layers), 2 biological filters (a trickling and a submerged biological filter) which had been activated beforehand (4 weeks prior to the experiment), and aeration systems present at the level of both rearing units and biological filters. The daily water exchange rate was maintained at 10%.

Within the experimental trial, 4 types of feeds were tested, including 1 commercial feed and 3 innovative feeds developed based on an alternative protein source represented by cricket meal (*Acheta domesticus*) and phytogenic additives as antioxidants: *Curcuma longa*—turmeric and *Beta vulgaris*—beetroot. Therefore, the following experimental variants were designed and the following feeds were administered: CF—commercial fish feed, IF—innovative feed with cricket meal, BIF—innovative feed with cricket meal and beetroot, and TIF—innovative feed with cricket meal and turmeric. The protein content was same in both commercial feeds, as well as in innovative feeds. The proximate composition of the innovative fish feeds was detailed in the patent application no. 00271/2021 submitted to the State Office for Inventions and Trademarks, Romania. A proximal composition of feed is presented in Table 1.

The feeds were administered two times a day (09:00 and 16:00). No issues related to a lack of appetite were encountered during the experimental period. In the first 22 days of the experiment, the feeding rate of 2% was calculated in relation to the initial biomass, while on the 23rd day, the total biomass weight was determined and the administrated feed quantity was adjusted by applying the 2% feeding rate in relation to the recently determined biomass weight value.

Throughout the experiment, water quality parameters were within the optimal range for koi carp rearing, according to Boyd and Tucker [63], as follows: temperature of 19.5–24.2 °C, 6–8.1 mg/L dissolved oxygen (DO), pH of 6.5–8.1 UpH, and 0.008–0.010 mg/L ammonium (NH_4_).

### 2.3. Amino Acid Analysis from the Innovative Feeds

The extraction and identification of free amino acids from the developed innovative fish feeds (IF, BIF, and TIF) were performed according to the method described by Oroian et al. [64]. Briefly, 15 mL of 15% trichloroacetic acid (TCA) was added to approximately 1 g of feed sample, after which the pH of the resulting mixture was adjusted to 2.2. Further, a volume of 25 mL of a 15% trichloroacetic acid solution was added to the extract. The supernatant was collected and filtered using a 0.45 µm microfilter. The determination of organic components was realized from a 100 µL volume of the filtered supernatant, using the EZfaast GC-MS kit (Phenomenex, Torrance, CA, USA). The reagents used are shown in Table 2. The extraction is performed via a packed sorbent tip that binds amino acids while allowing interfering compounds to flow through them. Amino acids on the sorbent are then extruded into the sample vial and quickly derivatized with the reagent at room temperature in aqueous solution.

The equipment used for the identification and separation of free amino acids was represented by a gas chromatograph coupled with a mass spectrometer (MS) equipped with a Zebron TM ZB-AAA column (10 m × 0.25 mm, film thickness: 0.25 μm) [64]. A total number of 27 amino acids (alanine, sarcosine, glycine, α-aminobutyric acid, valine, arginine, leucine, isoleucine, threonine, serine, proline, asparagine, aspartic acid, methionine, 3-hydroxyproline/4-hydroxyproline, phenylalanine, glutamic acid, α-aminoadipic acid, α-aminopimelic acid, glutamine, ornithine, glycyl-proline, hydroxylysine, histidine, lysine, tyrosine, and tryptophan) were determined from the samples. The results, expressed as μg/mg fish feed, were calculated based on the area of each “peak” and compared with the standard solution which consists of 33 amino acids (alanine, sarcosine, glycine, α-aminobutyric acid, valine, leucine, allo-isoleucine, isoleucine, threonine, serine, proline, asparagine, thioproline, aspartic acid, methionine, 3-hydroxyproline/4-hydroxyproline, phenylalanine, glutamic acid, α-aminoadipic acid, α-aminopimelic acid, glutamine, ornithine, glycyl-proline—2 isomers, proline-hydroxyproline, histidine, lysine, tyrosine, tryptophan, cystathionine, and cystine). The calibration curve was developed in the concentration rage 50 nmoles/m–200 nmoles/mL. Internal standard was used composed of β-amino isobutyric acid. All the determinations were carried out in triplicate.

Considering the replacement of protein from fish feeds using alternative sources (e.g., plants and insects), it is necessary to consider the content of essential amino acids due to their decisive role in the optimal development, growth, reproduction, and welfare status (immunity, antioxidative defense, etc.) of fish. The essential amino acids determined from the innovative fish feeds are as follows: arginine, histidine, isoleucine, leucine, lysine, methionine, phenylalanine, threonine, tryptophan, and valine. In the case of nonessential amino acids, the following were determined: alanine, asparagine, aspartate, glutamate, glycine, serine, and tyrosine.

The concentrations of essential and nonessential amino acids detected in each variant (IF, BIF, and TIF) are presented in Table 3.

### 2.4. Fish Growth Performance, Feed Utilization, and Condition Factor

Growth performance was evaluated based on the following indicators: individual biomass gain (IBG; g/fish), relative grow rate (RGR; g/g/day), specific grow rate (SGR; % fish biomass/day), feed conversion ratio (FCR; kg feed intake/kg fish biomass gain), protein efficiency ratio (PER; kg/kg), condition factor (K), and allometric growth [65]. These growth indicators were calculated by the following equations:

Individual biomass gain:

IBG = (Bf) − (Bi)/fish number [g/fish],(1)

where:

Bf—final fish biomass, and Bi—initial fish biomass.

Relative growth rate:

RGR = ((Bf − Bi)/t)/Bi [g/g/day],(2)

where:

Bf—final fish biomass; Bi—initial fish biomass, and t—duration of the experiment.

Specific growth rate:

SGR = 100 ∗ (ln Bf − ln Bi)/t [% fish biomass/day],(3)

where:

Bf—final fish biomass, Bi—initial fish biomass, and t—duration of the experiment.

Feed conversion ratio:

FCR = F/IBG [kg feed intake/kg fish biomass gain],(4)

where:

F—feed intake, and FBG—individual biomass gain.

Protein efficiency ratio:

PER = IBG/(F ∗ CP/100) [kg/kg],(5)

where:

IBG—individual biomass gain, F—feed intake, and CP—crude protein.

Fulton condition factor:

K = BW/TL^3^ × 100 [g/cm^3^], (6)

where:

BW—fish body weight (g), and TL—fish total length (cm).

Allometric growth:

W = aTL ^b^, (7)

where:

W—weight (g); TL—total length (cm); a—constant coefficient; and b—slope coefficient.

### 2.5. Fish Oxygen Consumption—Respirometry

Oxygen consumption (MO—mg O_2_/kg/h) was assessed through the intermittent-flow respirometry technique using four metabolic chambers, with a volume of 2.6 L each, from Loligo system (Loligo^®^ Systems, Viborg, Denmark). All the chambers were inserted into the same water tank so that the water temperature and quality fell in the same range. Also, in the tank, the water oxygen saturation was maintained at 95% by using an air compressor and two aeration stones.

The fish specimens were introduced into three metabolic chambers equipped with an oxygen port for the measurement of oxygen consumption. Inside the chambers, the water recirculation was periodically alternated from the flush phase (120 s) to the water mixing phase (waiting phase—60 s) and recirculation phase (measurement phase—240 s). Each metabolic chamber is equipped with two pumps to ensure a cycle of intermittent-flow respirometry (flush, waiting, and measurement phases).

The measurement of oxygen is carried out every second, and at the end of the cycle, the average value of oxygen is provided. Before conducting the trials, the oxygen sensors were calibrated using the sodium sulphite (Na_2_SO_3_) solution. A fourth metabolic chamber was used as a control variant (fish were not introduced into it) to measure the background respiration.

Data were obtained by using the AutoRespTM software (version 2.3.0) and the DAQ-M data acquisition equipment from Loligo^®^ Systems, Viborg, Denmark.

The measurement of oxygen consumption for each fish specimen (n = 9 for each experimental variant), was carried out for 24 h, and an average value was generated every 7 min. Before performing the measurements (24 h prior), no feed was administered to the fish biomass. The measurements were performed at the end of the experiment.

Therefore, AutoRespTM was used to calculate the metabolic rate (MO) based on the equation below:

MO_2_ = ([O_2_]in − [O_2_]out) × F/BW, (8)

where:

F = water flow rate (L/h);

[O_2_] in = oxygen content in water inflow (mg O_2_/L);

[O_2_] out = oxygen content in water outflow (mg O_2_/L);

BW = body weight of the fish used for the trial (kg).

Based on the obtained data, the standard metabolic rate (SMR—mgO_2_/kg/h), routine metabolic rate (RMR—mgO_2_/kg/h), maximum metabolic rate (MMR—mgO_2_/kg/h), and aerobic metabolic scope (AS—mgO_2_/kg/h) were determined.

SMR is an indicator used for the estimation of oxygen uptake rate by the fish that are in an inactive state (no locomotion or feeding). SMR is evaluated after an acclimation period (3 h) in the metabolic chamber [66,67].

RMR is an indicator that typically measures the average oxygen uptake rate necessary to estimate SMR over 24 h, following the protocol proposed by Chabot et al. [66].

MMR was obtained by applying the chase method described by Rosewarne et al. [68]. The fish specimens were chased until exhaustion in a circular tank, after which they were placed in a metabolic chamber (after the flush phase). Afterward, the highest value registered of metabolic rate during the recovery period represents the value of MMR [67,68].

AS represents the maximum capacity of the fish to supply oxygen necessary to sustain metabolic activities beyond SMR. It is a widely used indicator of the minimal rate of energy required to maintain life, and it is calculated by extracting the SMR from MMR [69].

### 2.6. Hematological Profile and Blood Biochemical Indicator Analysis

Blood samples were collected at the beginning and the end of the experiment, and no feed was administered 24 hr before the procedure. From each rearing unit, a statistically representative number of fish (n = 7) were randomly selected to be anesthetized with 2-phenoxyethanol. The blood samples were collected from the caudal vein using a 2.0 mL syringe. The blood samples were then transferred into previously heparinized Eppendorf tubes and analyzed immediately.

The red blood cell count (RBCc—cells/μL blood × 10^6^) was performed using a hemocytometer and Vulpian solution. The hemoglobin concentration (Hb—g/dL) was determined using Drabkin’s reagent and measured at 540 nm with a UV-VIS spectrometer. The hematocrit (PVC—%) was determined by using the centrifugation technique [70]. The erythrocyte constants (mean corpuscular volume (MCV—µm^3^), mean corpuscular hemoglobin (MCH—pg), and mean corpuscular hemoglobin concentration (MCHC—g/dL)) were calculated using a standard equation based on RBCc, Hb, and PVC, as described by Svobodova et al. [70] and Ispir et al. [71].

The blood biochemical indicators were measured from the blood plasma; thus, the blood samples were centrifugated, followed by the separation of the plasma from the blood cells. The analyzed biochemical parameters were as follows: albumin (g/dL), alkaline phosphatase (ALPs—U/L), alanine aminotransferase (TGP—U/L), aspartate aminotransferase (TGO—U/L), amylase (U/L), calcium (Ca—mg/dL), cholesterol (mg/dL), HDL-cholesterol (HDL—mg/dL), creatinine (mg/dL), lactate dehydrogenase (LDH—U/L), lipase (U/L), total bilirubin (TB—mg/dL), total protein (TP—g/dL), triglycerides (mg/dL), urea (mg/dL), and growth hormone (GH—mg/mL). All biochemical indicators were measured spectrophotometrically (colorimetric method) using commercial kits from Bionik (Tehran, Iran).

### 2.7. Oxidative Stress

The oxidative stress was evaluated based on the lipid peroxidation (MDA—nmol/mL) and total antioxidant capacity (TAC—mm Trolox), which were determined from the blood plasma. The lipid peroxidation was determined by measuring the malondialdehyde concentration in the presence of thiobarbituric acid, in accordance with Draper and Hadley [72]. The total antioxidant capacity was determined using ABTS-(2,2-azinobis 3-ethylbenzothiazoline-6sulphonic acid) in accordance with the method described by Van Den Berg [73].

### 2.8. Statistical Analysis

All the data registered following the measurements were analyzed using SPSS 21 (SPSS Inc.; Chicago, IL, USA) software. The values in the tables are presented as mean ± standard deviation. Data normality was evaluated using Shapiro–Wilk test. The one-way ANOVA test was employed to identify the differences between the experimental variants. Also, the ANOVA results were validated by using the Kruskal–Wallis test, which does not imply using the normality test since it does not assume a normal distribution of the residuals, unlike the analogous one-way analysis of variance—thus, no previous normality test was performed in this case. The Student’s *t*-test was used to compare initial data with final results in terms of hematological parameters and growth hormone indicator. Thus, a *p*-value lower than 0.05 was considered statistically significant. The length–weight relationships and allometric growth were determined using Microsoft Office 365 Excel software.

## 3. Results

### 3.1. Amino Acid Content in Innovative Fish Feeds

As can be observed in Table 3, the highest concentrations of essential amino acids were found in the case of the innovative feed with beetroot (BIF). The lowest concentrations were recorded predominantly in the case of the IF diet, except for the histidine, methionine, and tryptophan concentrations, which recorded the lowest values in the case of the TIF variant.

Regarding the non-essential amino acids, the highest values were recorded in the BIF diet, except for aspartic acid, glutamic acid, α-Aminoadipic acid, and 3-hydroxyproline/4-hydroxyproline, which registered the highest concentration in the TIF feed. Also, α-Aminopimelic acid and sarcosine recorded the highest values in the IF feed (Table 3).

### 3.2. Fish Growth Performance, Feed Utilization, and Condition Factor

**Figure 1:**
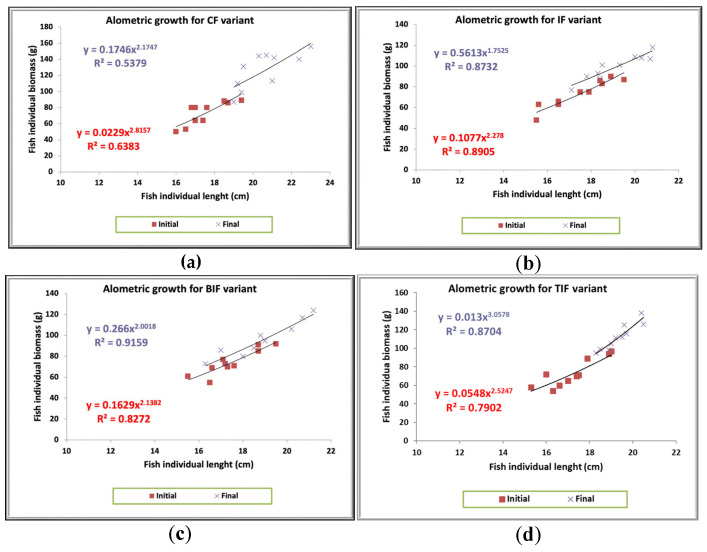
Representation of allometric growth in each experimental variant: (**a**)—CF variant, (**b**)—IF variant, (**c**)—BIF variant, (**d**)—TIF variant.

When it comes to fish growth performance, the commercial feed (CF) generated the highest biomass gain (Table 4). However, it did not register the best performance when it came to the Fulton coefficient and allometric coefficient (Table 4 and Figure 1). The condition factor (Fulton coefficient) is a well-known standard tool that is applied to identify the welfare of fish, while the allometric coefficient is used to identify possible deviations from the hypothetical growth of fish [74]. As can be observed in Table 4, the best fish growth status was identified in the variant to which the feed formulated with insect meal and turmeric powder was administered. Considering that Fulton coefficient registered the best results in the TIF variant, it can be speculated that the antioxidant effect of turmeric has increased the health and welfare status of the fish biomass. Obvious significant differences (*p* ˂ 0.05) between the insect formulated feeds (IF, BIF, and TIF) are encountered in terms of individual biomass gain, relative growth rate, specific growth rate, feed conversion ratio, and protein efficiency ratio, respectively, in the case of the TIF variant, to which turmeric was administered (Table 4).

As can be observed in Figure 1, the variant to which the commercial feed was administered (CF) registered a negative allometric growth (b < 3), and even a decrease in the “b” coefficient was highlighted by the end of the experimental period (b = 2.1747). The same trend was observed for the BIF and IF variants. The best performance in terms of fish allometric growth was obtained in the case of the TIF variant, in which positive allometry was observed (b > 3; b = 3.0578) (Figure 1). Monitoring the allometric factor is important for optimizing the feeding strategy by adjusting nutrient intake according to the fish growth pattern [75].

### 3.3. Fish Oxygen Consumption—Respirometry

Regarding the fish metabolic rate, the results registered for SMR, RMR, MMR, and AS for each experimental variant are presented in Table 5.

In the case of the SMR, a significant reduction was recorded in the IF variant (*p* ˂ 0.05) compared to the CF variant (18.17%), BIF variant (16.66%), and TIF variant (8.80%). At the same time, a significant increase in the SMR was found in the case of the administration of commercial feed (CF) (*p* ˂ 0.05) (9.16%) compared to the TIF variant (Table 5).

RMR registered a significant increase (*p* ˂ 0.05) of 20.64% in the CF, 20.06% in the TIF, and 26.08% in the BIF variant compared with the IF variant (Table 5). However, compared to the CF variant, the RMR reduction was not significant in the BIF and TIF variants (*p* ˃ 0.05) (Table 5).

Regarding the MMR, the highest value was registered in the case of the BIF variant and it was significantly higher (*p* ˂ 0.05) compared to the IF variant, CF variant, and TIF variant, respectively (Table 5). The value of the MMR registered in the IF variant was significantly lower (*p* ˂ 0.05) (7.49%) than the value obtained in the CF variant.

Regarding the AS, a significantly higher value (p ˂ 0.05) in the BIF experimental variant was registered compared to the IF variant (48.79%) and compared to the CF variant (42.04%) (Table 5). Even though the AS value recorded in the IF variant was lower compared to the one registered in the CF variant, between these experimental variants, there were no significant differences (*p* > 0.05).

### 3.4. Hematological Profile and Blood Biochemical Indices

**Figure 2:**
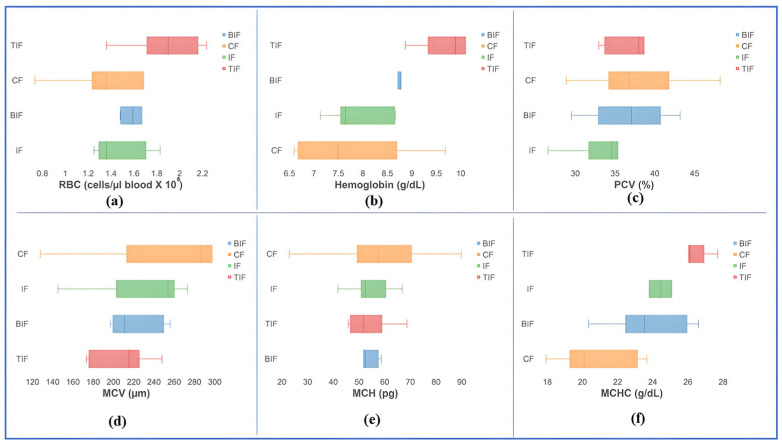
Hematological parameters at the beginning of the experimental trial: (**a**)—RBC level, (**b**) hemoglobin level, (**c**)—PCV content, (**d**)—MCV volume, (**e**)—MCH content, (**f**)—MCHC concentration.

At the beginning of the experiment, the hematological parameters of koi carp did not present high variability among the testing groups and registered no statistical differences (*p* > 0.05) among the experimental variants. For instance, RBCs were between 1.49 ± 0.23 cells/µL blood × 10^6^ for the IF variant and 1.87 ± 0.31 cells/µL blood × 10^6^ for the TIF variant (Figure 2). Hematocrit values were recorded between 32.72 ± 3.35% in the IF variant and 38.29 ± 5.42% in the TIF group (Figure 2). Hemoglobin values of 7.83 ± 1.20 g/dL in the CF variant and 9.95 ± 0.92 g/dL in the TIF variant were recorded (Figure 2).

**Figure 3:**
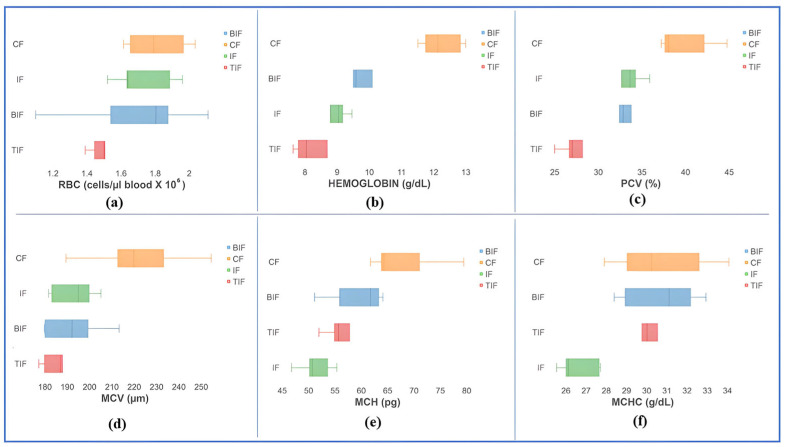
Hematological parameters at the end of the experimental trial. (**a**)—RBC level, (**b**) hemoglobin level, (**c**)—PCV content, (**d**)—MCV volume, (**e**)—MCH content, (**f**)—MCHC concentration.

It was observed that the commercial feed did not generate any changes in RBC concentrations throughout the experimental period and differences were not obtained (*p* > 0.05) in terms of RBC concentrations at the beginning of the experiment and end of the experiment. The same phenomenon was observed for the IF (*p* > 0.05), TIF (*p* > 0.05), and BIF (*p* > 0.05) in terms of RBC changes. Nevertheless, an increasing trend of RBC concentrations was observed from the beginning of the experiment to the end, with average values from 1.66 to 1.81 cells/µL blood × 106 in the CF variant, from 1.49 to 1.73 cells/µL blood × 10^6^ in the IF variant, and from 1.65 to 1.69 cells/µL blood × 10^6^ in the BIF variant. Interestingly, in the case of the TIF variant, the level of RBC concentration decreased from 1.87 cells/µL blood × 10^6^ at the beginning of the experiment to 1.51 cells/µL blood × 10^6^ at the end of the experiment (Figure 2 and Figure 3).

The hematocrit level did not change from the beginning of the experiment to the end in the case of the CF variant (*p* > 0.05), IF variant (*p* > 0.05), and BIF variant (*p* > 0.05). However, an increasing trend was registered from the beginning to the end of the experimental trial, from 37.96% to 39.96% in the CF variant and from 32.72% to 33.21% in the IF variant, respectively (Figure 2 and Figure 3). As in the case of the RBC concentration, it was observed that in the TIF variant, the hematocrit levels registered significant differences (*p* ˂ 0.05), with a decrease towards the end of the experiment, from 38.29% at the beginning of the experiment to 28.74% at the end. A decrease was also observed in the BIF variant for hematocrit levels, from 36.68% at the beginning of the experiment to 32.30% at the end of the experimental trial (Figure 2 and Figure 3).

The hemoglobin levels did not register significant differences (*p* > 0.05) between the beginning of the experiment and the end of the experiment in the case of the IF variant, TIF variant, and BIF variant, respectively. The CF variant registered strong significant differences (*p* ˂ 0.01) between the beginning and the end of the experiment, with a mean increase from 7.83 g/dL to 12.24 g/dL. A decrease in hemoglobin was observed in the case of the TIF variant, from 9.95 g/dL at the beginning of the experiment to 8.64 at the end of the experimental trial.

The mean corpuscular volume (MCV) did not register significant differences (*p* > 0.05) between the beginning of the experiment and the end of the experiment in the case of the CF variant, IF variant, TIF variant, and BIF variant, respectively. In all variants, a decreasing trend was observed between the beginning and end of the experimental trial, with mean values from 277.98 µm to 221.89 µm in the CF variant, from 226.96 µm to 192.90 µm in the IF variant, from 207.61 µm to 189.61 µm in the TIF variant, and from 222.66 µm to 192.89 µm in the BIF variant (Figure 2 and Figure 3).

The mean corpuscular hemoglobin (MCH) did not register significant differences (*p* > 0.05) between the beginning of the experiment and the end of the experiment in the case of the CF variant (*p* = 0.40), IF variant, TIF variant, and BIF variant, respectively.

The mean corpuscular hemoglobin concentration (MCHC) registered significant differences between the beginning and the end of the experiment in the CF variant (*p* ˂ 0.05), in TIF variant (*p* ˂ 0.01), and the BIF variant (*p* ˂ 0.01), respectively. An increase was registered between the beginning of the experiment and the end, with values from 20.82 g/dL to 30.77 g/dL in the CF variant, from 26.16 g/dL to 30.11 g/dL in the TIF variant, and from 23.78 g/dL to 30.71 g/dL in the BIF variant. No significant differences (*p* > 0.05) were obtained in the IF variant between the beginning and end of the experiment.

The results registered after the blood biochemical analysis are shown in Table 6.

Thus, as can be observed in Table 6, statistical differences in albumin, ALP, TGP, amylase, TGO, Ca, cholesterol, creatinine, HDL, LDH, lipase, TB, or TP between the variants were not registered (*p* > 0.05) at the end of the experiment.

Significant differences (*p* < 0.01) were registered in urea between the TIF and IF and between the TIF and CF, respectively, with the TIF having lower values compared to the rest of the variants (Table 6). Further, significant differences (*p* < 0.05) were observed between the BIF and CF and between the IF and CF, respectively, with higher values in the CF.

Significant differences were also registered in triglycerides between the TIF and CF (*p* < 0.01), IF and CF (*p* < 0.01), and BIF and CF, respectively (*p* < 0.05), with higher values in the CF variant.

**Figure 4:**
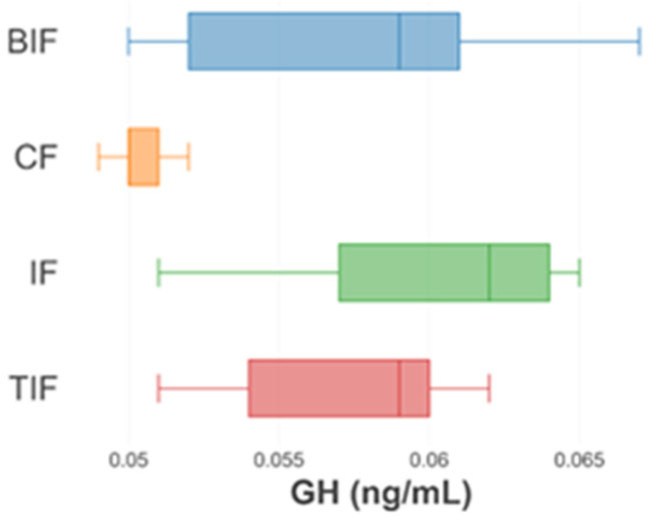
Growth hormone in experimental variants at the beginning of the experimental period.

**Figure 5:**
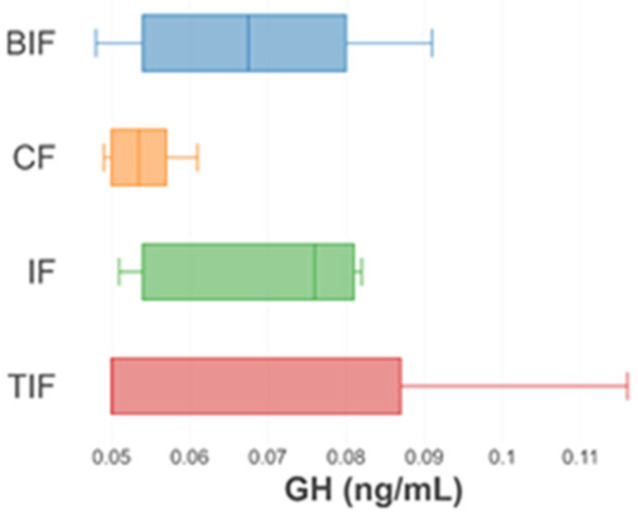
Growth hormone in experimental variants at the end of the experimental period.

The results for the growth hormone concentration (GH) obtained at the beginning and the end of the experiment are presented in Figure 4 and Figure 5, respectively.

At the beginning of the experiment, no significant differences (*p* > 0.05) were registered in the case of the GH in the experimental variants. The highest value was 0.059 ± 0.006 ng/mL, registered in the IF variant, and the lowest value was recorded in the CF variant (0.050 ± 0.001 ng/mL) (Figure 4).

At the end of the experiment, significant differences were obtained between the IF (0.070 ± 0.014 ng/mL) and CF (0.054 ± 0.005 ng/mL) variants (*p* < 0.05), with the GH being greater in the IF. The values recorded in the BIF and TIF variants were 0.068 ± 0.016 ng/mL and 0.067 ± 0.028 ng/mL, respectively, which are similar to the values obtained in the IF variant (Figure 5).

### 3.5. Oxidative Stress

The concentrations for the lipid peroxidation and total antioxidant capacity in the blood plasma of koi carp associated with each experimental variant are presented in Table 7. Thus, regarding MDA concentration, it was observed that between the experimental variants, there were no registered significant differences (*p* ˃ 0.05), but the lowest concentrations were recorded in the case of the TIF and IF variants, followed by the CF variant. The highest concentration of MDA was registered in the BIF variant, which was 13.66% higher compared to the CF variant, 20.93% higher compared to the IF variant, and 22.35% higher compared to the TIF variant, respectively.

The highest TAC concentrations were recorded in the case of the BIF and TIF variants, but no significant differences were recorded between the experimental variants (*p* ˃ 0.05). This aspect may be due to the content of antioxidants present in beetroot and turmeric, which can also be considered nutraceuticals, introduced in the fish diet from the BIF and TIF variants, respectively. The lowest TAC concentration was recorded in the IF variant, but it was not significantly (*p* ˃ 0.05) lower than the concentration recorded in the CF variant (Table 7).

## 4. Discussion

In 2017, at the level of the European Union, the use of insect meal in fish feed was approved by Regulation (EU) 2017/893. In this regulation, the seven insect species authorized to be used in fish feed were presented: black soldier fly (BSF)—*Hermetia illucens*; common housefly (HF)—*Musca domestica*; yellow mealworm (MW)—*Tenebrio molitor*; lesser mealworm—*Alphitobius diaperinus*; house cricket—*Acheta domesticus*; banded cricket—*Gryllodes sigillatus*; and field cricket—*Gryllus assimilis*) [20]. According to the circular economy concept, insects are worthy candidates for aquafeed ingredients. Thus, many countries with intensive aquaculture industries have started to replace fish meal with insect meal [76]. Sardine, anchovy, herring, capelin, mackerel, and other small pelagic marine fish species are sources of fish meal, but due to the gradual decline in wild stocks, the use of these sources is no longer sustainable [77,78,79,80]. Numerous studies have indicated the negative impact of insect meal used in fish diets due to its chitin content, which is known to reduce fish growth and is associated with reduced protein and lipid absorption at the gut level [81,82]. Nevertheless, more recent studies have demonstrated that chitin has prebiotic effects because it has the potential to stimulate the fermentation process in the gastro-intestinal tract, which, in the case of *Oncorhynchus mykiss,* contributes to improved intestinal health and general fish welfare [24,83], fish immunity [76,77], and disease resistance [30,84]. This has also been found in studies on shrimp—*Litopenaeus vannamei* [85] and *Cryphiops caementarius* [84]. Eggink et al. [86] found that the Nile tilapia and rainbow trout can digest 1.8%, 2.7%, and 15.4% chitin in dry matter feed. This can be potentially utilized as a nutrient source, but in low concentrations because the fish species studied could digest chitin but its digestibility decreased with higher dietary chitin inclusion levels. Moreover, Andriani et al. [87] suggest that the use of chitosan, which is a natural compound that can be obtained from chitin, in fish diets can improve feed digestibility, nutrient uptake, and the overall productivity of *Labeo rohita* species, which is a fish from the carp family (*Cyprinidae*), and tilapia. Considering the best performances in terms of Fulton and allometric coefficients, it seems that turmeric increases the overall digestibility of insect-based fish feeds and can be used as an ingredient to mitigate the limiting effects of insect meal (due to its chitin content). The capacity of turmeric to increase nutrient metabolism and digestibility, and thus growth performance in fish, has been previously highlighted [88,89,90]. This is a result of enhanced digestive proteolytic enzyme activity (especially lipase and trypsin) in the fish intestine [91]. Chitin is a common substrate of lipase which hydrolyzes oligosaccharides [92].

Different feeding trials have shown the growth of trout (*Oncorhynchus mykiss*) reared with different levels of *T. molitor* meal [93,94]. In a study conducted by Nogales-Merida et al., it was highlighted that the total replacement of fish meal with cricket (*Acheta domesticus*) meal in cyprinids’ diet covers the necessary amino acids range in order to ensure the optimum development of fish biomass [95]. Moreover, it was demonstrated that insect meal does not alter the sensory quality of fish meat [20,96]. Recent studies reveal that consumers exhibit generally positive attitudes towards and an acceptance of the utilization of insects in animal feed, particularly for fish and poultry [23,97,98]. Nevertheless, the selection of optimal insect species for the production of fish feed represents a considerable challenge. Insects such as crickets from the *Orthoptera* order were reported to have a high crude protein level ranging from 55 to 73%, as well as sufficient essential amino acids [32]. Thus, it has been concluded that cricket meal has the potential to partially or completely replace fish meal in fish feed [99]. Based on the results presented in Table 3, it is clear that the addition of turmeric and beetroot in fish feeds formulated with insect meal increases the concentration of essential and non-essential amino acids. This is due to the high concentration of some amino acids present in these phytoadditives. Moulick et al. [100] showed a higher concentration of amino acids in beetroot and turmeric, compared to carrot, which can play an important role in achieving nutritional balance for physiological processes to work properly in the body. Essential amino acids in fish feeds such as methionine, tryptophan, arginine, histidine, and lysine can improve digestive enzyme activity and thus increase nutrient digestibility and overall feed utilization [101,102,103,104,105,106,107]. At the same time, optimum levels of essential amino acids in fish feeds can mitigate physiological stress. For instance, tryptophan is not just involved in protein synthesis but is also the only precursor of serotonin and melatonin [103]. Non-essential amino acids such as alanine, aspargine, glutamine, proline, and serine also hold a nutritional role in fish diets because their presence is involved in the energy conservation necessary for protein synthesis [108].

It is well known that growth performance in aquaculture is directly correlated to fish welfare status. Hematological parameters are efficient instruments for evaluating fish welfare in response to stress and immune status [109,110,111]. The interventions of any stressors can induce hematological changes in fish, depending on the type and time of action [112]. On the other hand, it is known that fish welfare is greatly influenced by the administered feed [113]. Other studies have argued that the partial or total replacement in feed of fish meal with soybean [114,115], housefly maggot (*Musca domestica*) [116], and cricket (*Gryllus bimaculatus*) [32] does not lead to significant changes in terms of hematological parameters in different fish species.

In our case, the results obtained at the end of the experiment associated with the hematological parameters follow the reference range for cyprinids, namely between 0.33 and 2.95 cells/µL blood x 10^6^ for the RBC and 13.7–43.6% for the PCV, between 130.9 and 412.7 μm for the MCV concentration, 31.8 and 139 pg for the MCH concentration, and between 12 and 44.6 g/dL for the MCHC concentration [117]. The MCV, MCH, and MCHC are erythrocyte indices and express the size and hemoglobin content of erythrocytes. It was observed that including turmeric and beetroot in a mixture with insect meal in fish diets led to a reduction in MCV concentration, which led to an increase in MCHC concentration compared to the IF variant. But these differences in the reduction in the MCV concentration and the increase in the MCHC concentration are not validated from a statistical point of view because *p* ˃ 0.05 (Figure 3). This was also observed in the case of *Cyprinus carpio* species reared with a diet supplemented with neem (*Azadirachta indica*) [118].

Regarding the hemoglobin concentration in the case of the CF variant, a higher value (12.24 ± 0.58 g/dL) was registered, which is outside the normal range specified by Witeska et al. [117]: 3.76–11.43 g/dL. An increased Hb concentration in fish blood may indicate an increased demand for oxygen in tissues [119]. This may be due to the variability in energy demand at the level of the body and due to the metabolism activity necessary to maintain the vital functions of the organism. At the same time, it is also observed that the fish from the CF variant showed the highest oxygen consumption in respirometry tests (Table 5). It is well known that feed type and quality play a decisive role in animal husbandry because they directly influence the metabolic processes functioning within the organism. In our present study, we observed that the innovative feed based on insect meal led to a decrease in hemoglobin concentration, falling within the normal limits, and a reduction in oxygen consumption (Figure 2, Table 5).

The decrease in hematocrit levels in the IF, TIF, and BIF variants leads to an increased plasma level, a fact that improves electrolytes and protein transport in the bloodstream, contributing thus to ensuring the tissue’s need for oxygen. In the variants to which turmeric and beetroot were administered in combination with insect meal, a reduction in the number of erythrocytes was observed compared to the one which was not supplemented with antioxidants. Previously, it has been highlighted that curcumin has the potential to chelate and bind ferric iron (Fe^3+^), thus reducing iron bioavailability [120]. Since iron is essential for hemoglobin synthesis and red blood cell production, a reduction in available iron could lead to a lower erythrocyte count and hematocrit level. This was observed also in another study in which *Clarias gariepinus* was fed with a diet supplemented with 4% turmeric, and the fish specimens registered lower hematocrit levels compared to the control (0% turmeric) variant [53].

There was a decrease in the ALP concentration (*p* ˃ 0.05) in the variants in which the protein was replaced by insect meal and in the variants supplemented with turmeric and beetroot (respectively, the IF followed by the TIF and BIF), but the difference was not statistically significant (Table 6). However, we can speculate that this may indicate the better health status of their cellular membranes. Similar results were obtained in a study in which *Argyrosomus japonicus* was reared on a diet supplemented with *Ulva* sp. [121]. The ALP is a marker enzyme measured to assess the integrity of the plasma membrane and the endoplasmic reticulum [122].

Based on the results of the TGP and TGO enzymes as determined from the fish blood, the condition of the liver, kidneys, and gills is determined. An intense enzymatic activity leads to an increased TGO and TGP concentration [123], which leads to the degradation of these tissues. As observed in Table 5 and Table 6, regarding the TGO and TGP enzyme, creatinine, and the MDA concentration, the results obtained in all the experimental variants did not show statistically significant differences (*p* ˃ 0.05). At the end of the experiment, there was a slight increase in TGO, TGP, and creatinine concentration in the CF and BIF variants (*p* ˃ 0.05) (Table 6), which was correlated with the increase in MDA concentration (*p* ˃ 0.05) (Table 7). Therefore, supplementing the IF diet with turmeric (TIF variant) has the potential to reduce TGO and TGP enzymes, creatinine, and oxidative stress by reducing the lipid peroxidation index (MDA concentration) (Table 6 and Table 7), which can contribute to an improvement in health. For conclusive results from a statistical point of view, it is recommended to use these diets over a longer period than 8 weeks. Mohammed and Hasan [124] observed that the occurrence of oxidative stress in the common carp was correlated with an increase in TGO and TGP enzyme activity due to the permeability alterations in cellular membranes.

The highest albumin concentrations were registered in the case of the TIF variant, followed by the BIF and IF variants (*p* ˃ 0.05). The lowest concentration was recorded in the variant to which the commercial feed was administered (*p* ˃ 0.05). Even though no statistical differences were obtained between the experimental variants, this may show the synergic potential of feed additive turmeric and beetroot in combination with insect meal (the TIF and BIF variant) regarding the nutritional value of innovative feed for fish compared to the commercial feed (CF variant). Kulkarni [125] argues that the presence of albumin in fish blood indicates good health.

In the case of lipase, reduced enzymatic activity was observed in the variants to which the innovative feed was administrated compared to the commercial feed; however, the differences were not statistically significant (*p* ˃ 0.05). In the case of amylase, an increase in enzymatic activity was observed in the variants that received the innovative feed supplemented with turmeric (TIF) and beetroot (BIF). This indicates that supplementing insect-based fish feeds with these phytobiotics contributes to the nutritional value of the feed. Similar results were obtained in the case of *Labeo rohita* species reared using a feed based on alternative animal protein and garlic, which led to an increased amylase activity compared to the ones reared with a commercial feed [126]. This indicates that the bioactive compounds present in turmeric and beetroot can have the potential to mitigate the influence of chitin present in insect meal on the fish digestive tract, increasing the digestibility of the feed in the TIF and BIF variants. Nevertheless, the differences related to the amylase activity between the experimental variants were not significant (*p* ˃ 0.05), which means that although we obtained better results in the TIF and BIF variants, they are not statistically validated.

It seems that the innovative feed, as well as the one supplemented with turmeric and beetroot, has a beneficial effect on koi carp by its reducing cholesterol (*p* ˃ 0.05), HDL (*p* ˃ 0.05), triglycerides (*p* ˂ 0.05), and urea (*p* ˂ 0.05) and by increasing the concentration of calcium (*p* ˃ 0.05) in serum blood (Table 6). The observed changes suggest replacing fish meal with cricket meal (IF) has a potential positive influence on fish welfare, as well as supplementing innovative feed (IF) with turmeric (TIF) and beetroot (BIF).

Regarding the urea concentration in the blood plasma, similar results were found in other studies, in which it was found that bioactive compounds from different plants such as rosemary, thyme, and fenugreek did not lead to significant changes in the urea concentration from the blood plasma of *Dicentrarchus labrax* species [127], as well as in the case of paprika, oregano, and garlic administration in *Cyprinus carpio* diets [128]. In our study, the lowest levels of urea were registered in the TIF variant. Higher urea levels are associated with excessive protein breakdown, and it has been observed that curcumin manifests anti-catabolic effects, meaning it helps reduce protein degradation by modulating metabolic pathways [129]. At the same time, curcumin has the potential to enhance lipid metabolism by activating peroxisome proliferator-activated receptor alpha (PPAR-α). PPAR-α stimulation promotes lipid oxidation, which means triglycerides are broken down and used for energy instead of accumulating in the blood [130].

Regarding the TB concentration, it was observed in the case of the innovative feeds (IF, BIF, and TIF) developed based on cricket meal, turmeric, and beetroot, respectively, that the results fall within the optimum limits, respectively, 0.2–2 mg/dL [131]. However, the value obtained in the CF variant was not significantly lower compared to the rest of the variants.

In the case of TP concentration, a slight reduction was observed in the IF, TIF, and BIF variants. Nevertheless, the values obtained are within the optimal limits for koi carp [131].

The GH concentration recorded the highest value in the IF variant, followed by the BIF and TIF variants. Therefore, the addition of turmeric and beetroot increased the nutritional value of the innovative feed compared to the commercial one. Growth hormone is an indicator that underlines the growth capacity of fish by promoting cell division through the development of somatic cells [132,133].

Following the biochemical analysis of the blood samples, it can be stated that the addition of turmeric as well as beetroot in the fish feed based on insect meal (IF) lead to an overall improvement in the fish’s physiological state. Ashry et al. [88] drew similar conclusions about turmeric supplementation in *Sparus aurata* and Giri et al. [134] drew similar conclusions in *Cyprinus carpio*.

The changes in metabolic rate are associated with a secondary response to stress, which comprises various biochemical and physiological indicators [135].

When it comes to fish oxygen consumption, the highest values of the metabolic rate were recorded in the CF and BIF variants, in which the SMR, RMR, and MMR (Table 5) registered elevated values. This indicates that the fish consume more energy, thus leading to the need to administer more feed in order to obtain an optimum growth performance (especially in the case of the CF and BIF variants). The same aspect is highlighted by Peixoto et al. [136] in the case of perch reared on a diet supplemented with *Gracilaria* sp., which presented an increased RMR.

On the other hand, other researchers support the fact that the increased metabolism activity could be associated with the upregulation of specific and non-specific immune mechanisms [137,138,139]. According to Raberg et al. [140], the change in metabolic rate may be due to differential energy costs associated with the stimulation of innate or adaptive immune defenses.

Based on the results of the RMR and MMR, in our present study, the koi carp diet based on the alternative protein from cricket meal (IF) and the innovative feed supplemented with turmeric (TIF) lead to an improvement in fish oxygen consumption in stress conditions. In Table 4 and Table 6, it can be observed in the CF and BIF variants a potential correlation between the results of the lipid peroxidation indicator (MDA concentration) (*p* ˃ 0.05) and the oxygen consumption, as well as the results regarding the metabolic rate (SMR and RMR) (*p* ˃ 0.05). Both in the case of the lipid peroxidation index analysis and in the case of the SMR and RMR measurements, the results obtained in the CF and BIF variants were higher than those obtained in the IF variant, with the specification that at the level of oxidative stress (MDA concentration), the results are not statistically validated. This was also noted by Peixoto et al. [141]. Rudneva and Shaida [142] support the fact that the production of reactive oxygen species (ROS) increases with the increase in the energy requirement of muscle cells. On the other hand, metabolic stress is associated with the depletion of cellular ATP because the energetic costs of the organism tend to increase due to the activation of various cell defense components (Hsps, antioxidant enzymes, MFO, etc.) [142]. It is well documented that antioxidant enzymes play a key role in the inactivation of ROS and thereby maintain oxidative stress at low levels [142].

Even though the present study presents extensive information and different experimental layers on the potential use of cricket meal and phytogenic additives such as turmeric and beetroot for the production of koi carp, it is important to acknowledge the following limitations:

1. A relatively small sample size—This sample size number may be considered adequate for preliminary insights. However, a larger sample can minimize variability. Future studies could consider using a larger fish population to improve the level of confidence in our results;
2. Species specificity—The results obtained within this study should be extrapolated cautiously for other species due to differences in species feeding ecologies, nutritional requirements, development stages, lifecycles, and rearing systems.

## 5. Conclusions

As a general conclusion of the present study, we observed that the feed based on cricket meal led to (29%) higher growth performances in fish compared to the commercial feed, based on the growth hormone analysis.

Supplementation with turmeric in aquafeeds based on cricket meal promoted fish growth performance even further (by 5%) by generating a higher Fulton coefficient and a higher allometric factor (>3) compared to the commercial feed.

The hematological and biochemical analysis revealed that the feed based on cricket meal and turmeric generated lower levels of urea (26% lower), cholesterol (11% lower), and triglycerides (26% lower) in fish blood compared to the commercial feed. At the same time, it promoted feed digestibility due to the higher value for amylase activity (>7%).

Further, the innovative fish feed based on cricket meal and turmeric has the potential to mitigate oxidative stress, due to the malondialdehyde concentration manifested in the fish biomass.

The use of cricket meal and turmeric in the diet of koi carp positively influenced the oxygen consumption due to having the highest registered SMR and RMR values.

Nevertheless, the relatively small size of the sampled population approached within the present study limits the extrapolation of our results to larger fish populations.

Considering the above-mentioned points, it is recommended to use aquafeeds based on cricket meal and turmeric for other fish species (especially marine and carnivorous species) to close the knowledge gap related to this subject.

The present study contributes to the sustainable intensification of the aquaculture industry by reducing the environmental footprint and impact of fish meal.

## 6. Patents

This work is based on the patent application A/00271 from 21 May 2021 submitted to the Romanian State Office for Inventions and Trademarks.

## Figures and Tables

**Table 1:** Proximal composition of feed for each experimental variant.

Component	CF	IF	BIF	TIF
Crude protein (%)	38.00	38.00	38.00	38.00
Crude fat (%)	10.00	17.74	17.92	18.15
Total carbohydrate (%)	10.00	16.55	17.04	18.00

**Table 2:** Reagents in the EZfaast kit.

Reagent	Ingredients	Volume
Reagent 1 Internal Standard Solution	Norvaline 0.2 mM N-propanol 10%	50 mL
Reagent 2 Sodium Carbonate Solution	Na_2_CO_3_	90 mL
Reagent 3A Eluting Medium Component I	Sodium Hydroxide	60 mL
Reagent 3B Eluting Medium Component II	3-Picoline N-propanol	40 mL
Regent 4 Organic Solution I	Propyl-chloroformate Chloroform	4 vials, 6 mL each
Reagent 5 Organic Solution II	Iso-octane	50 mL
Reagent 6 Acid Solution	Hydrochloric Acid 1N	50 mL
SD Amino Acid Standard Mixtures	AA Standard Mixture	2 vials of each SD, 2 mL each

**Table 3:** The concentrations of essential and nonessential amino acids in the innovative fish feeds (μg/g).

Amino Acids	CF	IF	BIF	TIF
Histidine ^e^	203.10 ^a^	168.36 ^a^	198.38 ^a^	124.5 ^b^
Lysine ^e^	396.12 ^b^	308.24 ^b^	622.98 ^a^	520.35 ^a^
Isoleucine ^e^	315.09 ^a^	146.82 ^b^	302.29 ^a^	219.21 ^b^
Leucine ^e^	418.63 ^a^	115.63 ^b^	220.64 ^b^	181.72 ^b^
Methionine ^e^	141.16 ^a^	129.62 ^a^	116.52 ^a^	116.03 ^a^
Phenylalanine ^e^	296.31 ^a^	146.79 ^b^	224.74 ^a^	167.6 ^b^
Threonine ^e^	187.11 ^a^	110.95 ^a^	171.65 ^a^	129.88 ^a^
Tryptophan ^e^	129.83 ^a^	136.63 ^a^	148.41 ^a^	135.26 ^a^
Valine ^e^	439.16 ^a^	246.99 ^b^	489.82 ^a^	469.78 ^a^
Arginine ^e^	601.72 ^b^	409.07 ^c^	758.76 ^a^	642.41 ^b^
Alanine ^n^	397.21 ^c^	353.68 ^c^	710.35 ^a^	593.33 ^b^
Asparagine ^n^	145.90 ^b^	125.98 ^b^	183.51 ^a^	159.27 ^b^
Aspartic acid ^n^	907.04 ^c^	998.40 ^c^	1521.40 ^b^	2532.00 ^a^
Glutamic acid ^n^	1219.27 ^b^	1354.60 ^b^	1761.20 ^b^	3575.10 ^a^
Glutamine ^n^	386.14 ^c^	676.10 ^b^	1008.00 ^a^	448.30 ^c^
Glycine ^n^	320.47 ^a^	200.18 ^b^	407.84 ^a^	0
Serine ^n^	279.06 ^b^	237.66 ^b^	456.97 ^a^	229.49 ^b^
Tyrosine ^n^	481.09 ^a^	142.05 ^b^	204.89 ^b^	170.74 ^b^
α-Aminoadipic acid ^n^	79.02 ^a^	75.00 ^a^	82.00 ^a^	110.81 ^a^
α-Aminopimelic acid ^n^	1652.81 ^a^	2003.3 ^a^	1883.50 ^a^	1983.20 ^a^
3-hydroxyproline/4-hydroxyproline ^n^	89.12 ^a^	94.00 ^a^	97.52 ^a^	103.17 ^a^
Proline ^n^	284.60 ^a^	189.71 ^b^	352.90 ^a^	277.49 ^a^
Hydroxylysine (2 isomers) ^n^	91.82 ^a^	105.05 ^a^	128.42 ^a^	109.85 ^a^
Sarcosine ^n^	0	30.06 ^a^	0	0
α-Aminobutyric acid ^n^	0	49.00 ^a^	52.00 ^a^	0
Ornithine ^n^	0	0	69.91 ^a^	0
Glycyl-proline (dipeptide) ^n^	0	0	298.53 ^a^	0

Note: ^e^ = essential amino acids; ^n^ = non-essential amino acids. Values with different letters in a row indicate significant differences (ANOVA, *p* < 0.05) among experimental variants.

**Table 4:** Growth performance indicators of koi carp reared with different types of feeds.

Technological Indicators	CF	IF	BIF	TIF
Initial stocking density (kg/m^3^)	6.67 ^a^	6.69 ^a^	6.76 ^a^	6.64 ^a^
Initial average fish weight (g)	73.4 ^a^	73.6 ^a^	74.4 ^a^	73.0 ^a^
Final stocking density (kg/m^3^)	11.52 ^a^	9.02 ^b^	8.56 ^b^	10.23 ^a^
Final average fish weight (g)	126.7 ^a^	99.2 ^b^	94.2 ^b^	112.5 ^a^
Individual biomass gain (g)	53.3 ^a^	25.6 ^c^	19.8 ^c^	39.5 ^b^
Relative growth rate (g/g/day)	0.013 ^a^	0.006 ^c^	0.005 ^c^	0.010 ^b^
Specific growth rate (%/day)	1.01 ^a^	0.55 ^c^	0.44 ^c^	0.80 ^b^
Feed conversion ratio (g/g)	1.49 ^c^	3.11 ^a^	4.06 ^a^	2.00 ^b^
Protein efficiency ratio (g/g)	1.77 ^a^	0.85 ^c^	0.65 ^c^	1.32 ^b^
Fulton coefficient	1.46 ^b^	1.42 ^b^	1.44 ^b^	1.54 ^a^

Note: Values with different letters in a row indicate significant differences (ANOVA, *p* < 0.05) among experimental variants.

**Table 5:** Fish metabolic rate recorded at the end of the experiment.

Experimental Variant		CF	IF	BIF	TIF
SMR (mg O_2_/kg/h)	Mean ± Stdv.	150.60 ± 8.26 ^a^	123.23 ± 10.99 ^c^	147.86 ± 20.51 ^a^	135.12 ± 14.25 ^b^
RMR (mg O_2_/kg/h)	Mean ± Stdv.	162.57 ± 8.02 ^a^	132.71 ± 10.59 ^b^	161.18 ± 21.85 ^a^	159.41 ± 31.83 ^a^
MMR (mg O_2_/kg/h)	Mean ± Stdv.	337.24 ± 46.13 ^b^	301.41 ± 30.32 ^c^	412.98 ± 88.35 ^a^	351.33 ± 58.63 ^b^
AS (mg O_2_/kg/h)	Mean ± Stdv.	186.65 ± 51.60 ^b^	178.18 ± 37.63 ^b^	265.12 ± 69.17 ^a^	216.21 ± 50.50 ^b^

Note: SMR—standard metabolic rate; RMR—routine metabolic rate; MMR—maximum metabolic rate; AS—aerobic metabolic scope. Values with different letters in a row indicate significant differences (ANOVA, *p* < 0.05) among experimental variants.

**Table 6:** Blood biochemical parameter concentrations.

Biochemical Parameter		Experimental Variants			
		CF	IF	BIF	TIF
Albumin (g/dL)	Mean ± Stdv.	1.05 ± 0.09 ^a^	1.12 ± 0.09 ^a^	1.13 ± 0.16 ^a^	1.16 ± 0.14 ^a^
ALPs (U/L)	Mean ± Stdv.	9.77 ± 0.45 ^a^	9.45 ± 0.46 ^a^	9.14 ± 0.22 ^a^	9.38 ± 0.55 ^a^
TGP (U/L)	Mean ± Stdv.	6.78 ± 0.20 ^a^	6.74 ± 0.11 ^a^	6.77 ± 0.17 ^a^	6.62 ± 0.34 ^a^
TGO (U/L)	Mean ± Stdv.	264.47 ± 47.72 ^a^	235.98 ± 126.06 ^a^	284.03 ± 99.92 ^a^	212.79 ± 43.21 ^a^
Amylase (U/L)	Mean ± Stdv.	26.58 ± 6.23 ^a^	23.85 ± 4.03 ^a^	28.03 ± 8.45 ^a^	28.31 ± 7.15 ^a^
Lipase (U/L)	Mean ± Stdv.	14.81 ± 1.64 ^a^	14.28 ± 1.53 ^a^	14.72 ± 0.89 ^a^	14.01 ± 1.76 ^a^
Ca (mg/dL)	Mean ± Stdv.	9.28 ± 0.36 ^a^	9.50 ± 0.22 ^a^	9.54 ± 0.39 ^a^	9.32 ± 0.34 ^a^
Cholesterol (mg/dL)	Mean ± Stdv.	204.14 ± 26.96 ^a^	174.74 ± 33.26 ^a^	177.32 ± 13.53 ^a^	180.44 ± 27.51 ^a^
HDL (mg/dL)	Mean ± Stdv.	66.38 ± 10.74 ^a^	59.26 ± 8.15 ^a^	60.84 ± 7.89 ^a^	59.93 ± 9.17 ^a^
Triglycerides (mg/dL)	Mean ± Stdv.	308.23 ± 41.79 ^a^	212.84 ± 56.96 ^b^	247.57 ± 53.50 ^b^	225.91 ± 15.94 ^b^
Creatinine (mg/dL)	Mean ± Stdv.	0.39 ± 0.16 ^a^	0.26 ± 0.11 ^a^	0.34 ± 0.14 ^a^	0.25 ± 0.11 ^a^
Urea (mg/dL)	Mean ± Stdv.	1.01 ± 0.08 ^a^	0.82 ± 0.06 ^a^	0.80 ± 0.07 ^b^	0.74 ± 0.06 ^b^
LDH (mg/dL)	Mean ± Stdv.	455.29 ± 70.23 ^a^	419.17 ± 216.79 ^a^	417.00 ± 249.86 ^a^	385.47 ± 264.35 ^a^
TB (mg/dL)	Mean ± Stdv.	0.17 ± 0.02 ^a^	0.43 ± 0.20 ^a^	0.38 ± 0.14 ^a^	0.32 ± 0.19 ^a^
TP (g/dL)	Mean ± Stdv.	2.32 ± 0.18 ^a^	2.17 ± 0.11 ^a^	2.26 ± 0.23 ^a^	2.24 ± 0.17 ^a^

Note: ALPs—Alkaline phosphatase; TGP—Alanine aminotransferase; TGO—Aspartate aminotransferase; Ca—calcium; HDL—High-density lipoprotein—cholesterol; LDH—Lactate dehydrogenase; TB—Total bilirubin; TP—Total protein. Values with different letters in a row indicate significant differences (ANOVA, *p* < 0.05) among experimental variants.

**Table 7:** The oxidative stress parameters recorded at the end of the experiment.

Experimental Variant		CF	IF	BIF	TIF
MDA (nmol/mL)	Mean ± Stdv.	1.83 ± 0.11 ^a^	1.72 ± 0.11 ^a^	2.08 ± 0.40 ^a^	1.70 ± 0.03 ^a^
	Min.	1.71	1.62	1.72	1.67
	Max.	1.94	1.84	2.51	1.74
TAC (mM Trolox)	Mean ± Stdv.	3.69 ± 0.88 ^a^	3.39 ± 0.42 ^a^	4.04 ± 0.39 ^a^	3.83 ± 0.16 ^a^
	Min.	3.16	2.92	3.61	3.68
	Max.	4.70	3.74	4.38	4.00

Note: MDA—lipid peroxidation; TAC—total antioxidant capacity. Values with different letters in a row indicate significant differences (ANOVA, *p* < 0.05) among experimental variants.

## Data Availability

Data will be made available on request.
